# Population differentiation and dynamics of five pioneer species of *Gaultheria* from the secondary forests in subtropical China

**DOI:** 10.1186/s12870-024-05189-z

**Published:** 2024-06-08

**Authors:** Yi-Rong Li, Peter W. Fritsch, Gui-Gang Zhao, Xiao-Juan Cheng, Zhao-Li Ding, Lu Lu

**Affiliations:** 1 School of Pharmaceutical Sciences, Yunnan Key Laboratory of Pharmacology for Natural Products, Yunnan College of Modern Biomedical Industry, Kunming Medical University, Kunming, 650500 Yunnan China; 2 Botanical Research Institute of Texas, 1700 University Drive, Fort Worth, TX 76017 USA; 3 Genome Center of Biodiversity, Kunming Institute of Zoology, Chinese Academy of Science, Kunming, 650223 China; 4 Yunnan Key Laboratory of Biodiversity Information, Kunming Institute of Zoology, Chinese Academy of Science, Kunming, 650223 China

**Keywords:** *Gaultheria*, Genetic structure, Isolation by distance, Isolation by environment, Pioneer species, Secondary forest, Subtropical China

## Abstract

**Background:**

The influence of native secondary succession associated with anthropogenic disturbance on the biodiversity of the forests in subtropical China remains uncertain. In particular, the evolutionary response of small understory shrubs, particularly pioneer species inhabiting continuously disturbed habitats, to topographic heterogeneity and climate change is poorly understood. This study aimed to address this knowledge gap by focusing on the *Gaultheria crenulata* group, a clade of small pioneer shrubs in subtropical China.

**Results:**

We examined the genetic structure and demographic history of all five species of the *G. crenulata* group with two maternally inherited chloroplast DNA (cpDNA) fragments and two biparentally inherited low-copy nuclear genes (LCG) over 89 natural populations. We found that the genetic differentiation of this group was influenced by the geomorphological boundary between different regions of China in association with Quaternary climatic events. Despite low overall genetic diversity, we observed an isolation-by-distance (IBD) pattern at a regional scale, rather than isolation-by-environment (IBE), which was attributed to ongoing human disturbance in the region.

**Conclusion:**

Our findings suggest that the genetic structure of the *G. crenulata* group reflects the interplay of geological topography, historical climates, and anthropogenic disturbance during the Pliocene–Pleistocene-Holocene periods in subtropical China. The observed IBD pattern, particularly prominent in western China, highlights the role of limited dispersal and gene flow, possibly influenced by physical barriers or decreased connectivity over geographic distance. Furthermore, the east-to-west trend of gene flow, potentially facilitated by the East Asian monsoon system, underscores the complex interplay of biotic and abiotic factors shaping the genetic dynamics of pioneer species in subtropical China’s secondary forests. These findings can be used to assess the impact of environmental changes on the adaptation and persistence of biodiversity in subtropical forest ecosystems.

**Supplementary Information:**

The online version contains supplementary material available at 10.1186/s12870-024-05189-z.

## Background

Subtropical China, is recognized as one of the world’s most species-rich forested regions [1]. Its ecosystems significantly contribute to biodiversity preservation and helps to maintain equilibrium of the global carbon cycle [2]. Occupying the region south of the Qinling-Huaihe River Line, north of the Leizhou Peninsula, and east of the Hengduan Mountains (i.e., 22–34°N, 98°E to the coastline in the east [3]), this area encompasses the main southern parts of China’s second-step and third-step terrain regions. The second step is characterized by plateaus and basins ranging from 1,000 to 2,000 m above sea level (a.s.l.), whereas the third-step comprises plains and hills below 500 m a.s.l. [4]. The forests in subtropical China have a highly heterogeneous topography and complex environmental history, with intense mountain uplifts and Pliocene–Pleistocene climatic dynamics, harboring refugia for many ancient lineages [5].

Subtropical China’s forests have undergone significant transformation into plantations and secondary forests due to prolonged and persistent anthropogenic disturbance, with some areas being replaced by densely populated agricultural lands [6, 7]. The influence of these secondary forests on the biodiversity of subtropical China has been a topic of long-standing debate in community ecology [8, 9]. They have been posited to decrease biodiversity [10], yet also have been thought to serve as biodiversity reservoirs [11]. Research on these forests has predominantly focused on ecosystem stability, species composition, and community/stand structure for ecosystem restoration [10, 12]. Less emphasis has been placed on adaptation to disturbance and successional dynamics of pioneer species in this ecosystem, particularly within the context of phylogeography at a geological scale. Secondary succession can affect genetic diversity [13], and so studying the population genetic dynamics of pioneer plant species may provide valuable insights into succession [14].

Population genetic studies on pioneer species in the secondary forests of the subtropical China have concentrated on widespread and dominant trees or large shrubs such as *Quercus fabri* Hance [15] and *Liquidambar formosana* Hance [16]. In contrast, short/small pioneer shrub species of the forest understory in subtropical China have largely been neglected. Although small understory shrubs are not the dominant element of the forests in subtropical China, their functional traits, activities, succession rate, and phylogenetic diversity in areas under secondary succession have been found to be influenced more strongly by topographic heterogeneity or microhabitats (i.e., more likely to escape extreme cold) than by changing climates [5, 17–19]. Conversely, other studies have found that such shrubs are strongly responsive to recent climate change [20, 21].

The *Gaultheria crenulata* group is a clade of five species of small understory shrubs, including *G. crenulata* Kurz, *G. luchunensis* Yi R. Li, Lu Lu & P.W. Fritsch, *G. pingbienensis* (C.Y. Wu ex T.Z. Xu) Yi R. Li, Lu Lu & P.W. Fritsch, *G. wuliangshanensis* Yi R. Li, Lu Lu & P.W. Fritsch, and *G. mangshanensis* Yi R. Li, Lu Lu & P.W. Fritsch [22]. The species of the group are some of the earliest pioneer colonizers in the secondary coniferous mixed forests in subtropical China. *G. crenulata* is the most widespread species of Chinese *Gaultheria*, occurring nearly throughout the area of China south of the Yangtze River Basin, and ranging from 200 to 3300 m elevation [22]. Because of the rich presence of methyl salicylate (oil of wintergreen; [23], this species is used in Chinese traditional medicine by > ten minorities [24] and which now is suffering heavy local harvesting. This is leading to the reduction of populations or individuals, as observed by us in the field. The *G. crenulata* group diverged earlier in the phylogenetic history of *Gaultheria* than most other Chinese species of the genus, and its crown age is estimated as ca. 4.1 Ma in the early Pliocene [25]. Thus, the genetic differentiation and demographic dynamics of the group can be expected to provide insights into the impacts of topography and climates from the Pliocene to present time on species adaptations in subtropical China. This in turn will aid understanding as to how these pioneer plants evolved in current secondary forests.

Here we combined geographic, environmental, and genetic data with a phylogeographic approach to uncover the genetic structure and dynamics of the *Gaultheria crenulata* group. We address the following questions on the secondary forests of the subtropical China. (1) In subtropical China, what is the pattern and strength of genetic differentiation of the group? (2) How did the group respond to long-term climatic and topographic dynamics? Does isolation by distance (IBD) or isolation by environment (IBE) better explain its genetic differentiation? (3) How have variations in topography and climate through time influenced the genetic differentiation and demographic history of the group?

## Results

### Genetic diversity and differentiation statistics

For the data from low-copy nuclear genes (LCG), the combined sequence totaled 1,442 bp in length, with *AAT*(aspartate aminotransferase) 670 bp and *LOC* (far1-related region) 772 bp. We identified 292 polymorphic sites, including 10 singleton variable sites and 282 parsimony-informative sites. The allelic richness (*A*_R_) of the whole dataset was 542 and nucleotide diversity (*π*) was 7.67 × 10^–3^. Genetic variation differs among populations, with *π* ranging from 0.00 to 9.95 × 10^–3^ and *A*_R_ ranging from 1 to 1.98 (Table 1). The highest level of genetic diversity was found in the SB population (*A*_R_ = 1.97, *π* = 9.95 × 10^–3^). Significant genealogically geographic structure was found in the overall (population) dataset (*N*_ST_ = 0.35 > *G*_ST_ = 0.19; *P* < 0.01). The combined length of the two chloroplast DNA (cpDNA) fragments was 1,268 bp, comprising *rpl*33-*psaJ* at 669 bp and *trnL*-*rpl*32 at 599 bp. We identified 29 polymorphic sites, consisting of 11 singleton variable sites and 18 parsimony-informative sites. The concatenation analysis based on cpDNA data revealed an overall haplotype diversity (*H*_d_) of 0.31 and nucleotide diversity *(π*) of 1.26 × 10–3 across 89 populations. Based on the PERMUT analysis of cpDNA data, the *Gaultheria crenulata* group revealed low to moderate genetic variation among populations at the species level (*H*_T_ = 0.34) and low genetic variation within populations (*H*_S_ = 0.091).Genetic differentiation incorporating distances among haplotypes (*N*_ST_ = 0.813) was found to be significantly higher than population differentiation without incorporating distance (*G*_ST_ = 0.739, *P* < 0.05).

**Table 1:** Sample location, sample size and genetic diversity of two cpDNA and two LCG (low-copy nuclear genes) loci, and cpDNA haplotypes in 89 sampled populations of the *G. crenulata* group. *H*d, cpDNA haplotype richness; *π*, nucleotide diversity; *A*_R_, nuclear sequence allelic richness

Region	Population	Location	Latitude	Longitude	cpDNA				LCG		
					n	Hd	π (× 10^–3^)	Haplotypes	n	*A*_R_	*π* (× 10^–3^)
The western China group	YHQ	Tenchong, Houqiao, YN, CN	25.36	98.25	17	0.00	0.00	H2(17)	17	1.76	2.80
	TC	Yawushan, Tengchong, YN, CN	25.43	98.50	13	0.15	0.12	H2(12), H19(1)	13	1.49	3.09
	YWX	Weixi, YN, CN	27.97	99.06	21	0.00	0.00	H2(21)	21	1.32	0.22
	YYD	Yongde, YN, CN	24.17	99.66	16	0.00	0	H2(16)	16	1.95	7.83
	BS	Changning, Baoshan, YN, CN	25.04	99.84	15	0.47	0.96	H2(11), H4(2), H5(1), H6(1)	15	1.54	1.58
	DL	Cangshan, Dali, YN, CN	25.81	100.11	22	0.50	1.9	H1(15), H2(5), H6(1), H7(1)	22	1.91	6.94
	YNJ	Dali, Nanjian, YN, CN	24.87	100.58	15	0.00	0.00	H1(15)	15	1.97	7.83
	JD	Jingdong, YN, CN	24.36	100.75	11	0.00	0.00	H13(11)	11	1.31	0.22
	SB	Shuangbai, Chuxiong, YN, CN	24.69	101.63	20	0.10	0.08	H1(19), H18(1)	20	1.97	9.95
	YYJ	Yuanjiang, YN, CN	23.66	101.77	9	0.00	0.00	H2(9)	9	1.90	7.57
	WD	Wuding, YN, CN	25.47	102.15	9	0.00	0.00	H1(9)	9	1.88	4.94
	XC	Lushan, SC, CN	27.83	102.26	20	0.00	0.00	H1(20)	20	1.83	4.55
	YHT	Yuxi, Hongta, YN, CN	24.38	102.32	17	0.00	0.00	H1(17)	17	1.98	8.64
	AN	Anning, YN, CN	24.65	102.34	10	0.38	1.3	H1(8), H2(1), H3(1)	10	1.73	5.20
	DQS	Daqingshan, SC, CN	27.75	102.34	14	0.00	0.00	H1(14)	14	1.93	7.53
	YLC	Luchun, YN, CN	22.99	102.39	14	0.65	2.14	H1(1), H2(2), H22(8), H23(3)	14	1.59	7.29
	XS	Xishan, Kunming, YN, CN	25.06	102.62	11	0.67	2.77	H1(3), H2(6), H20(1), H21(1)	11	1.94	6.03
	SGL	Liangshan, Ganluo, SC, CN	28.90	102.66	14	0.00	0.00	H1(14)	14	1.90	3.57
	SHD	Liangshan, Huidong, SC, CN	26.67	102.85	17	0.65	2.88	H1(9), H2(4), H2(4)	17	1.96	7.51
	YSM	Kunming, Songming, YN, CN	25.36	102.98	15	0.42	2.04	H1(11), H2(4)	15	1.95	7.64
	QJ	Yaoshan, Qiaojia, YN, CN	27.01	103.24	9	0.69	2.61	H1(5), H2(2), H16(1), H17(1)	9	1.88	4.04
	EMS	Emeishan, SC, CN	29.59	103.37	1	0.00	0.00	H1(1)	1	1.00	0.00
	YHZ	Huize, Qujing, YN, CN	26.52	103.45	17	0.00	0.00	H2(17)	17	1.98	6.56
	PB	Pingbian, YN, CN	22.97	103.69	8	0.25	0.2	H2(7), H9(1)	8	1.73	6.13
	DWS	Daweishan, Pingbian, YN, CN	22.93	103.69	11	0.00	0.00	H4(11)	11	1.75	3.29
	ZY	Zhanyi, Qujing, YN, CN	25.72	103.95	18	0.11	0.09	H2(17), H9(1)	18	1.95	7.47
	FY	Fuyuan, YN, CN	25.14	104.14	16	0.13	0.61	H1(1), H2(15)	16	1.96	7.72
	LJS	Wenshan, Laojunshan, YN, CN	23.23	104.18	10	0.00	0.00	H2(10)	10	1.94	8.30
	GZS	Zhushi, GZ, CN	27.96	104.66	10	0.00	0.00	H2(10)	10	1.95	7.79
	MLP	Wenshan, Malipo, YN, CN	23.17	104.74	12	0.00	0.00	H2(12)	12	1.98	8.65
	LPS	Liupanshui, GZ, CN	26.45	104.77	20	0.00	0.00	H2(20)	20	1.84	5.68
	HZ	Hezhang, GZ, CN	27.30	104.79	18	0.00	0.00	H2(18)	18	1.97	8.52
	ZX	Zhenxiong, YN, CN	27.41	104.83	13	0.00	0.00	H2(13)	13	1.97	7.05
	GDH	Xingren, GZ, CN	25.11	104.83	5	0.00	0.00	H2(5)	5	1.51	1.64
	YGN	Wenshan, Guangnan, YN, CN	23.69	104.85	14	0.00	0.00	H2(14)	14	1.98	8.87
	XR	Xingren, GZ, CN	25.45	105.38	17	0.00	0.00	H2(17)	17	1.95	8.14
	XNP	Baise, Napo, GX, CN	23.32	105.80	11	0.00	0.00	H2(11)	11	1.87	4.86
	CS	Changshun, GZ, CN	26.01	106.36	23	0.00	0.00	H2(23)	23	1.92	6.90
	GBS	Baise, Leye, Gantian, GX, CN	24.58	106.50	16	0.00	0.00	H2(16)	16	1.84	6.37
	GY	Guiyang, GZ, CN	26.28	106.63	12	0.17	0.13	H2(11), H9(1)	12	1.93	6.81
	GLL	Longli, GZ, CN	26.40	106.91	18	0.00	0.00	H2(18)	18	1.91	7.70
	DY14	Duyun, GZ, CN	25.88	107.09	27	0.00	0.00	H2(27)	27	1.92	6.49
	GPZ	Pingtang, GZ, CN	26.07	107.09	24	0.00	0.00	H2(24)	24	1.84	4.73
	GD	Guiding, GZ, CN	26.43	107.16	25	0.45	0.37	H2(17), H9(8)	25	1.93	6.13
	JFS	Chongqin, Jingfoshan, CQ, CN	29.08	107.21	18	0.00	0.00	H2(18)	18	1.87	4.28
	DY13	Duyun, GZ, CN	26.34	107.49	21	0.57	0.49	H2(11), H8(9), H9(1)	21	1.91	6.29
	YS	Shiqian, GZ, CN	27.40	108.05	29	0.00	0.00	H2(29)	29	1.91	5.64
	WXC	Leishan, GZ, CN	26.38	108.11	21	0.00	0.00	H2(21)	21	1.83	4.53
	SWT	Shiqian, GZ, CN	27.47	108.20	18	0.11	0.09	H2(17), H9(1)	18	1.93	7.81
	LS	Leishan, GZ, CN	26.37	108.28	27	0.00	0.00	H2(27)	27	1.95	5.99
	JWS	Jiuwanshan, Liuzhou, GX, CN	25.20	108.57	16	0.13	0.20	H2(15), H15(1)	16	1.94	6.67
	YW	Yingjiang, GZ, CN	27.90	108.61	14	0.00	0.00	H2(14)	14	1.90	4.78
	YJ	Yuanjiang, YN, CN	27.95	108.61	10	0.00	0.00	H2(10)	10	1.72	4.59
	YHA	Yuanjiang, YN, CN	27.95	108.61	10	0.00	0.00	H2(10)	10	1.57	3.79
	HLF	Laifen, Dahe, HB, CN	29.46	109.11	20	0.00	0.00	H2(20)	20	1.42	2.16
	YBS	Yuanbaoshan, GX, CN	25.38	109.13	15	0.00	0.00	H2(15)	15	1.95	6.15
	NXX	Xiangx, Huayuan, HN, CN	28.42	109.50	14	0.00	0.00	H2(14)	14	1.92	6.76
	NZJ	Huaihua, Zhijiang, HN, CN	27.54	109.56	18	0.00	0.00	H2(18)	18	1.97	9.03
	NJZ	Huaihua, Jingzhou HN, CN	26.80	109.63	18	0.00	0.00	H2(18)	18	1.90	6.70
	HMD	Lichuan, Moudao HB, CN	30.44	108.67	21	0.00	0.00	H2(21)	21	1.80	5.02
Total summary	60	NA	NA	NA	945	0.40	1.61	NA	945	459.0	8.21
The eastern China group	HMB	Lichuan, Maoba, HB, CN	30.06	109.06	20	0.00	0.00	H2(20)	20	1.00	0.00
	QFJ	Fengji, Zhuyuan,CQ, CN	31.25	109.29	18	0.00	0.00	H2(18)	18	1.11	0.08
	NTD	Huaihua, Tongdaoxian, Wanfoshan, HN, CN	26.30	109.92	19	0.00	0.00	H2(19)	19	1.78	3.69
	LG	Lingui, GX, CN	25.54	109.99	17	0.00	0.00	H2(17)	17	1.80	3.41
	NHJ	Huaihua, Hongjiang, HN, CN	27.19	110.18	14	0.00	0.00	H2(14)	14	1.86	6.58
	DYS	Dayaoshan, GX, CN	24.07	110.23	13	0.15	1.00	H2(12), H10(1)	13	1.70	2.87
	NCB	Shaoyang, Chenbu, HN, CN	26.35	110.28	21	0.00	0.00	H2(21)	21	1.74	2.95
	NWG	Shaoyang, Wugang, Yunshan, HN, CN	26.68	110.65	18	0.00	0.00	H2(18)	18	1.94	5.98
	DYDS	Yunfu, Luoding, Yadoushan, GD, CN	22.61	111.20	6	0.00	0.00	H2(6)	6	1.67	3.97
	DPL	Yongzhou, Daoxian, Dupangling, HN, CN	25.57	111.39	16	0.00	0.00	H2(16)	16	1.64	3.78
	YMS	Yongzhou, Shuangpai, Yangmingshang, HN, CN	26.01	111.92	21	0.00	0.00	H2(21)	21	1.78	3.99
	DLS	Lianzhou, Lianshan, Hedongcun, GD, CN	24.80	112.06	18	0.00	0.00	H2(18)	18	1.74	3.29
	DLN	Lianzhou, Liannan, Daping, GD, CN	24.65	112.19	18	0.00	0.00	H2(18)	18	1.78	4.36
	NMS	Chengzhou, Yizhang, Mangshan, HN, CN	24.95	112.97	14	0.14	0.93	H2(1), H10(13)	14	1.83	6.43
	TJS	Shoguan, Luoyang, Tianjingshan, GD, CN	24.70	113.02	14	0.14	0.12	H2(13), H14(1)	14	1.66	3.60
	NL	Shaoguan, Ruyuan, Nanling, GD, CN	24.91	113.05	15	0.00	0.00	H2(15)	15	1.60	3.35
	DLC	Shaoguan, Lechang, Wushan, GD, CN	25.37	113.51	19	0.00	0.00	H2(19)	19	1.82	5.62
	NGD	Chengzhou, Guidong, Shatian, HN, CN	25.87	113.80	20	0.00	0.00	H2(20)	20	1.46	2.20
	DWY	Shaoguan, Wengyuan, Wengcheng, GD, CN	24.41	113.91	2	0.00	0.00	H2(2)	2	1.00	0.00
	DNX	Shaoguan, Nanxiong, Baishun, Dongken, GD, CN	25.17	114.06	15	0.00	0.00	H2(15)	15	1.67	2.45
	JSC	Jian, Suichuan, Dafen, JX, CN	26.24	114.15	21	0.00	0.00	H2(21)	21	1.58	1.93
	JGS	Jian, Jinggangshan, Lanhuaping, JX, CN	26.57	114.18	19	0.11	0.09	H2(18), H14(1)	19	1.50	1.89
	JLN	Ganzhou, Longnan, Jiulianshan, JX, CN	26.56	114.50	20	0.10	0.08	H2(19), H14(1)	20	1.88	6.16
	DHY	Heyuanshi, Liyuan, Qutan, Xianzitan, GD, CN	24.49	114.74	18	0.00	0.00	H2(18)	18	1.89	7.54
	JXW	Ganzhou, Xunwu, Xiangshanzen, JX, CN	24.92	115.84	17	0.22	0.18	H2(15), H14(2)	17	1.32	1.17
	FMHS	Longyan, Liancheng, Juxizhen, Meihuashan, FJ, CN	25.46	116.79	21	0.10	0.46	H2(20), H11(1)	21	1.51	3.53
	FGT	Longyan, Gutian, Buyun, FJ, CN	25.26	116.86	20	0.10	0.16	H2(19), H12(1)	20	1.73	3.59
	FDQS	Zhangzhou, Jiufeng, Daqinshan, FJ, CN	24.22	117.10	17	0.12	0.57	H2(16), H11(1)	17	1.12	0.33
	FHJS	Longyan, Xingluo, Hongjianshan, FJ, CN	25.05	117.16	19	0.11	0.17	H2(18), H12(1)	19	1.68	3.29
Total summary	29	NA	NA	NA	490	0.09	0.43	NA	490	111.0	4.93
Total (all)	89	NA	NA	NA	1435	0.31	1.26	NA	1435	542.0	7.67

*Abbreviations: CN* China, *YN* Yunnan, *GZ* Guizhou, *GX* Guangxi, *SC* Sichuan, *CQ* Chongqing, *HB* Hubei, *HN* Hunan, *JX* Jiangxi, *GD* Guangdong, *FJ* Fujian

### Population genetic structure and migration

**Fig. 1:**
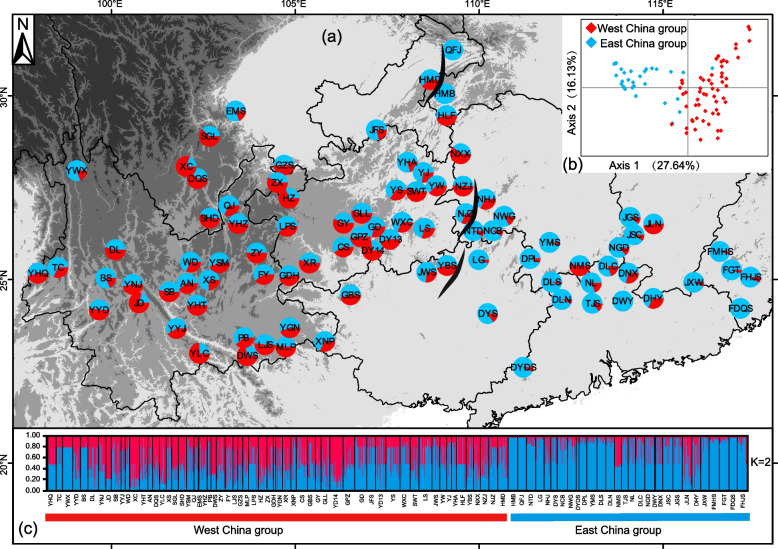
**a** Geographical distribution of the genetic clusters in 89 populations sampled from the *G. crenulata* group in subtropical China. Two genetic clusters are generated from LCG data (red/blue, and blue) with STRUCTURE analysis, K = 2 (see population codes in Table 1). The black dashed line represents the boundary that generally divides into two clusters (the WC group and the EC group), which also roughly corresponds to the boundary between the second-step region and the third-step region of China’s terrain; **b** Principal component analysis of LCG data of 1,435 individuals from 89 populations of the two lineages; **c** Admixture assignment for 89 populations with K = 2: each bar represents a population, with different colors corresponding to one of the ancestries, which was determined to be the optimal K value with the ΔK method. (Map source: https://www.webmap.cn/main.do?method=index)

For the LCG data, Bayesian clustering analysis with STRUCTURE indicated that the sequential increase in K values from 2 to 10 (see Fig. S1) provided into the relative strength of different signals of population subdivision within the *G. crenulata* group. The ΔK value was highest when K = 2, followed by K = 8, K = 3, and K = 5. In our analysis at K = 2, the data from the 89 populations revealed a significant divide along the Wushan and Xuefeng Mountains, which form the boundary between the second and third-step regions of China (Fig. 1). The western China group (WC, 60 populations) and eastern China group (EC, 29 populations) were genetically divided and exhibited a mosaic of clusters in the WC group (Table 1; Fig. 1). When K = 3, the results were consistent with K = 2. At K = 5, *G. wuliangshanensis* (JD in Fig. S1) and *G. luchunensis* (YLC in Fig. S1) separated from the WC group, forming a separate subpopulation, whereas *G. mangshanensis* (NMS in Fig. S1) separated from the EC group, forming another distinct subpopulation. At K = 8, *G. pingbienensis* separated from the WC group, forming a new subpopulation (DWS in Fig. S1). PCoA revealed distinct genetic structure among populations within the *G. crenulata* group, with the first and second axes capturing 27.6% and 16.1% of the total genetic variation, respectively. The WC group (shown in red) and EC group (shown in blue) exhibited distinct clusters, indicating their differentiation (Fig. 1).

**Fig. 2:**
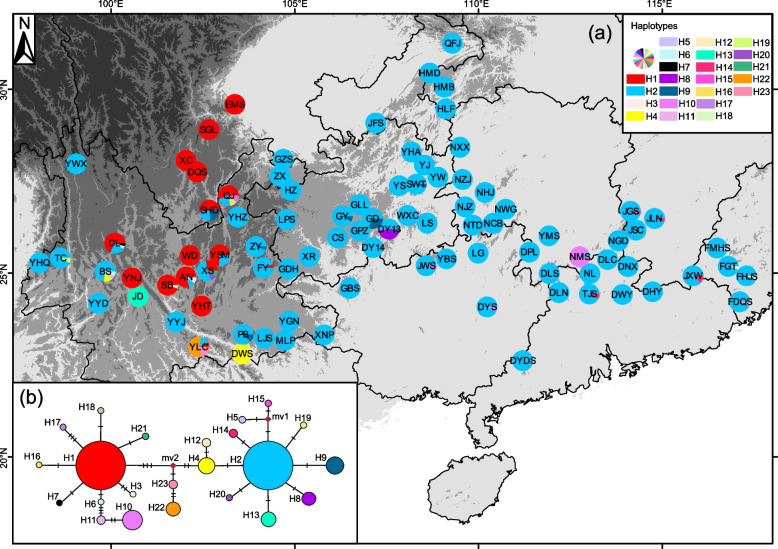
**a** Geographic distribution of the cpDNA haplotypes in 89 populations sampled from the *G. crenulata* group in subtropical China (see population codes in Table 1); **b** Network plot of the 23 cpDNA haplotypes recovered, where each haplotype is depicted with the same color as in the geographic distribution. The size of each circle corresponds to the frequency of each haplotype. The red diamonds on the branches represent hypothetical missing haplotypes. (Map source: https://www.webmap.cn/main.do?method=index)

Between the two cpDNA fragments combined, 23 haplotypes were obtained, with haplotypes H1 (in central and northern Yunnan and southern Sichuan) and H2 (in the remaining regions) accounting for two major components (Fig. 2). In the network analysis, 23 chloroplast haplotypes separated into two major groups. The first group consisted of 12 haplotypes (H3, H6, H7, H10, H11, H16, H17, H18, H21, H22, and H23) with H1 as the center, which then diverged in a star-like pattern to form a lineage. The second group comprised 11 haplotypes (H4, H5, H8, H9, H12, H13, H14, H15, H19, and H20) with H2 as the center, which also differentiated in a star-like pattern to form another lineage (Fig. 2). The SplitsTree analysis recovered *G. wuliangshanensis*, *G. pingbienensis*, *G. luchunensis*, and *G. mangshanensis* as forming sister groups distinct from *G. crenulata* (Fig. S2). The Bayesian search strategy-based analyses with MIGRATE for the LCG data indicated that long-term gene flow between populations is asymmetric, with a greater east-to-west tendency (EC group Θi = 0.097 vs. WC group Θi = 0.006; Nm (EC to WC) = 88.87 >  > Nm (WC to EC) = 2.94; S. M. Table S1).

In the AMOVA analyses, for the LCG data 14.45% of the variation was found between the WC group and the EC group. For each of the two groups, 27.0% was attributed to among-populations variation and 58.6% within-population variation (F_CT_ = 0.144, F_ST_ = 0.414, *P* < 0.01; Table 2). For cpDNA data, 5.5% of the variation was found between the two groups, 75.8% among populations within a group, and 18.7% within populations (F_CT_ = 0.055, F_ST_ = 0.814, *P* < 0.01; Table 2).

**Table 2:** Analysis of molecular variance (AMOVA) within the *G. crenulata* group based on cpDNA and LCG data

Region	Source of variation	d.f	Sum of squares	Variance components	Percentage of variation (%)	Fixation index
LCG	Among groups	1	1540.845	1.132 Va	14.45	F_CT_ = 0.144*
	Among populations within groups	86	6308.392	2.114 Vb	26.98	F_SC_ = 0.315*
	Within populations	1346	12,763.565	4.591 Vc	58.57	F_ST_ = 0.414*
	Total	1433	20,612.802	7.838		
cpDNA	Among groups	1	52.255	0.058 Va	5.53	F_CT_ = 0.055*
	Among populations within groups	86	1138.420	0.802 Vb	75.82	F_SC_ = 0.802*
	Within populations	1346	265.516	0.197 Vc	18.65	F_ST_ = 0.814*
	Total	1433	1456.192	1.060		

*d. f.* Freedom

^*^0.01 < *p* < 0.05

### Demographic history and divergence time estimates

For the LCG data, the expansion model was not rejected by either the SSD or the HRag statistics (*P* > 0.05, Table S2). The neutral tests revealed an overall selection effect across populations (Tajima’s *D* =  − 1.8803, *P* < 0.05; Fu and Li’s D* = 3.7249, *P* < 0.05). With the two groups analyzed separately, only the WC group exhibited significant values (Tajima’s *D* =  − 1.7758, *P* < 0.05; Fu and Li’s D* = 3.4592, *P* < 0.05), and mismatch analysis supported the historical population expansion model (both *P* > 0.05; Table S2). For cpDNA data, both the SSD and HRag indices did not reject the expansion model (*P* > 0.05; Table S2). However, the neutral test for the total populations showed significance (Tajima’s *D* =  − 1.3975, *P* < 0.05; Fu and Li’s D* =  − 3.8003, *P* < 0.05). With the two groups analyzed separately, the WC group exhibited a non-significant value of Tajima’s *D* =  − 0.9904 (*P* > 0.05). However, the value of Fu and Li’s D* =  − 4.1559 (*P* < 0.05) was significant, suggesting population expansion. The neutral tests of the EC group were insignificant (Tajima’s *D* =  − 1.2438, *P* > 0.05; Fu and Li’s D* = 1.2038, *P* > 0.05; Table S2).

**Fig. 3:**
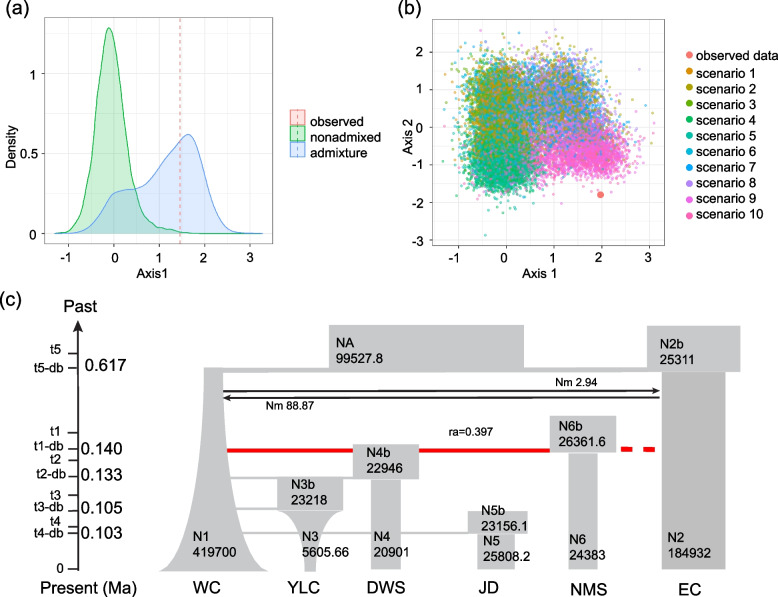
**a** Projection of the LCG dataset on a single LDA axis for the training set when analyzing the two hypotheses of scenarios (nonadmixed and admixture); **b** Projection of the LCG dataset on the first two LDA axes when analyzing the ten scenarios separately. Each colored point corresponds to a single simulation of one of the models; the red dot corresponds to the observed data. The observed data fall approximately within the range of the output of the conformity model on Axis 1 but fall outside of the range on Axis 2; **c** The best demographic model of divergence for the six classified groups in the *G. crenulata* group. Each block represents a current or ancestral population and its estimated effective population size. Arrows indicate the direction of gene flow, and the estimated migration rate is marked above or below the arrow. The timing of the splitting events is indicated in million years ago (Ma). The admixture ratio is indicated by the additional parameters “ra” and “1-ra,” representing the genetic contribution of the ancestral population Pop 1 and Pop 2, respectively

The DIYABC Random Forest analysis provided clear evidence of admixture within the *Gaultheria crenulata* group (Fig. 3). When the ten scenarios are analyzed separately, the observed data fall approximately within the range of the output of the conformity model on Axis 1 but fall outside of the range on Axis 2 (Fig. 3). The highest classification votes and estimated posterior probabilities showed that scenario 9, i.e., the ancient differentiation with recent mixing model, has the highest posterior probability (0.444) among all hypothetical scenarios (Table S3-S5). This suggests that the WC and EC groups diverged from a most recent common ancestor (NA). The *G. mangshanensis* population was identified as a genetically admixed population of the WC and EC groups. The time of divergence between the WC group and the EC group was estimated as 0.617 Ma. Demographic bottlenecks occurred ca. 5,340 generations ago (corresponding to roughly 26.7 kya, with the average five-year reproductive maturity period for *Gaultheria* considered [26]. These bottleneck events occurred prior to the divergence of *G. pingbienensis* (DWS), *G. luchunensis* (YLC), *G. wuliangshanensis* (JD), and *G. mangshanensis* (NMS) during the interval from 0.103 Ma to 0.140 Ma (Tables S3-S6, Fig. 3).

**Fig. 4:**
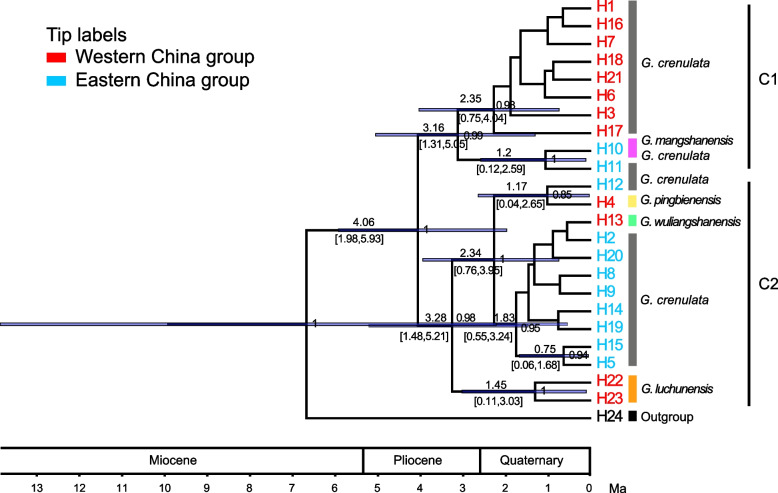
Chronogram of the *G. crenulata* group based on cpDNA data. The times in both best ages (in bold) and their 95% HPD with timescale blue bars (font in smaller size within brackets) are presented above the branches and posterior probability below the branches (only the divergence times for the nodes with posterior probability > 50% are shown)

From the BEAST results of cpDNA haplotypes, the estimated crown age of the *G. crenulata* group was inferred as early Pliocene (4.06 Ma, 95% HPD: 1.98–5.93 Ma, PP = 1.00). The haplotypes form two major subclades (C1 and C2; Fig. 4). The divergence time estimate for subclade C1 is 3.16 Ma (95% HPD: 1.31–5.05 Ma, PP = 0.99). This subclade is composed of haplotypes from most populations of the WC group and two of the EC group (NMS of *G. mangshanensis* and DWS, haplotypes H10 and H11, with divergence at ca. 1.2 Ma, 95% HPD: 0.12–2.59 Ma, PP = 1.00). The divergence time estimate for subclade C2 was 3.28 Ma (95% HPD: 1.48–5.21 Ma, PP = 0.98). This subclade is composed of haplotypes from most populations of the EC group and all private haplotypes. The private haplotypes H22 and H23 of *G. luchunensis* (YLC) form a sister group that diverged ca. 1.45 Ma (95% HPD: 0.11–3.03 Ma, PP = 1.00). Haplotype H4, exclusive to *G. pingbienensis* (DWS), diverged ca. 1.17 Ma (95% HPD: 0.04–2.65 Ma, PP = 0.85).

### IBD and IBE analyses

The MMRR analysis and Mantel analysis for both the LCG and cpDNA data showed a significant IBD (*P* < 0.01) and non-significant IBE (*P* > 0.05) across the overall (populations). When the WC group and EC group were tested separately, a positive IBD correlation was detected only in the WC group (*P* < 0.01; Table 3).

**Table 3:** Multiple matrix regression with randomization (MMRR) analysis and Mantel test results showing the relative contribution of isolation by spatial distance (IBD) and environment (IBE) with respect to multiple population genetic distance indices of six datasets: 1. LCG data of the overall populations, 2. cpDNA data of the overall populations, 3. LCG data of populations from the western China group, 4. cpDNA data of populations from the western China group, 5. LCG data of populations from the eastern China group, 6. cpDNA data of populations from the eastern China group

Region	Genetic divergence	randomization (MMRR)		Mantel tests	
		βIBD	βIBE	R^2^IBD	R^2^IBE
Overall LCG	F_ST_	27.0068**	-1.5669	0.1626**	0.0001
	F_ST_(1-F_ST_)	6.4882**	-0.8966	0.0109**	0.0001
Overall cpDNA	F_ST_	10.3392**	-0.5253	0.0259**	0.0000
	D_XY_	9.1954**	-0.8082	0.0207**	0.0000
WC LCG	F_ST_	10.5505**	-2.3759	0.0584**	0.0040
	F_ST_(1-F_ST_)	4.4366**	-2.0445	0.0110**	0.0027
WC cpDNA	F_ST_	7.6941**	1.0214	0.0335**	0.0005
	D_XY_	6.1723**	0.3871	0.0217**	0.0001
EC LCG	F_ST_	1.3072	0.8429	0.0072	0.0029
	F_ST_(1-F_ST_)	10.5505	-2.3759	0.0004	0.0043
EC cpDNA	F_ST_	-2.7227	1.5806	0.0181	0.0024
	D_XY_	-2.6533	1.4986	0.0148	0.0031

*Abbreviations: WC* The western China group, *EC* The eastern China group

**p < 0.01

### Ecological niche modeling

**Fig. 5:**
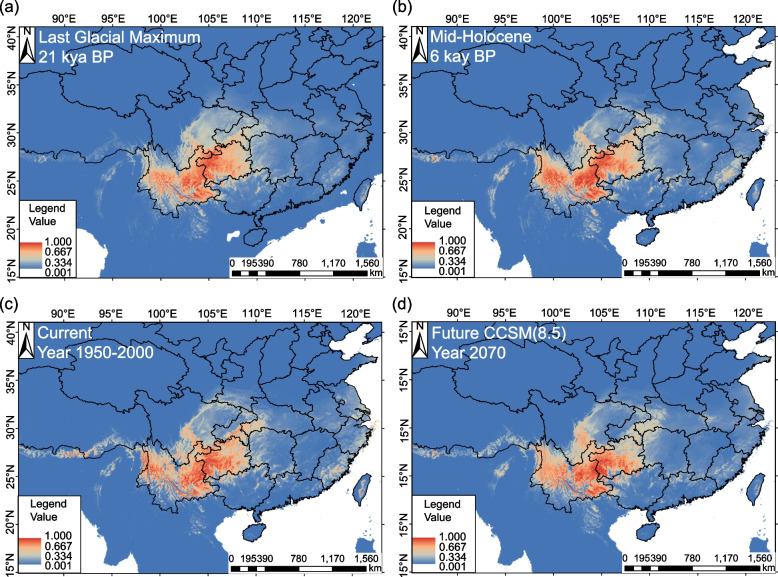
The predicted distribution of the WC group of the *G. crenulata* group in each of four climate scenarios A–D. **a** Last glacial maximum (LGM) based on the output of CCSM4; **b** Mid-Holocene (MH) based on the output of CCSM4; **c** present (1950–2000) based on the output of CCSM4; **d** future (2070) based on the output of CCSM4. (Map source: https://www.webmap.cn/main.do?method=index)

**Fig. 6:**
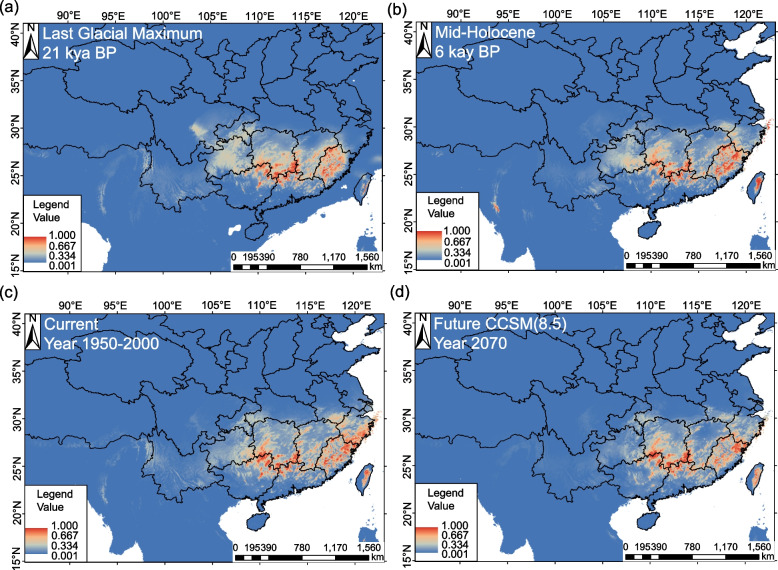
The predicted distribution of the EC group of the *G. crenulata* group in each of four climate scenarios A–D. **a** Last glacial maximum (LGM) based on the output of CCSM4; **b** Mid-Holocene (MH) based on the output of CCSM4; **c** present (1950–2000) based on the output of CCSM4; **d** future (2070) based on the output of CCSM4. (Map source: https://www.webmap.cn/main.do?method=index)

The average test AUC for replicate runs was 0.968 (SD = 0.010) for the WC group and 0.988 (SD = 0.004) for the EC group, indicating satisfactory model performance. The jackknife test revealed that the variable Bio14 (Precipitation of Driest Month) contributed most to the potential distribution modeling of the WC group (33.9%) and EC group both (68.3%), followed by Bio1 (Annual Mean Temperature) at 20.0% of the WC group and Bio5 (Max Temperature of Warmest Month) at 8.9% of the EC group (Table S7). The current distribution projections accurately depict the optimal areas for the *G. crenulata* group in the subtropical mountainous regions of China, spanning from northwestern to southeastern Yunnan, Guizhou, the border regions of Hunan, Guangdong, and Guangxi, and southeastern Fujian. During the LIG and MH periods, the potential range was slightly larger but less optimal in northwestern Yunnan. The WC group’s potential range during these periods was larger but also less optimal in northwestern Yunnan and Southern Sichuan. For the EC group, the potential range during the MH period was relatively smaller and more scattered, expanding from then to the present. Under the maximum greenhouse gas emission model of RCP 8.5 scenario, the overall geographic distribution is expected to shrink, with a significant reduction in optimal areas such as Chongqing, Hubei, and Northern Guizhou of the WC group (Fig. 5). The EC group, which is more heavily affected by anthropogenic disturbances, exhibits a more limited and fragmented optimal distribution area (Fig. 6).

## Discussion

### The biogeographic boundaries and chloroplast-nuclear discordance

Our LCG data revealed a significant divide along the Wushan and Xuefeng Mountains, which coincides with the boundary between the second- and third-step regions of China. This finding aligns with the east–west differentiation observed in many plant groups in subtropical China, e.g., *Quercus acutissima* Carruth. and *Toxicodendron vernicifluum* (Stokes) F. Barkley [4, 27]. However, for cpDNA data, a biogeographic division of the *G. crenulata* group into two lineages was observed: one from the high-elevation region within the second-step region of China (reaching nearly 2,000 m) in central and northern Yunnan and southern Sichuan, mainly characterized by subalpine mountains and mid-elevation basins with fragmented regions isolated by valleys (e.g., Jinsha River, Dadu River, Yalong River, and Anning River), the other from the lower-elevation area (ca. 1,000 m), characterized by gentle undulating hills and covering most of the eastern Yungui Plateau and the middle-lower reaches of the Yangtze River in the third-step plain (Fig. 2).

This contrast between nuclear and cpDNA data serves as evidence of how different genomes respond to past population fluctuations and biogeographic events [28] and highlights the influence of historical factors and geographical barriers in shaping the genetic structure of the *G. crenulata* group. The biogeographic nuclear-chloroplast discordance detected here has been observed in various other plant species. For example, mountain barriers led to chloroplast differentiation whereas nuclear gene flow remained extensive in *Ulmus lamellosa* C.Wang & S.L.Chang [29]. The distinct geographic structure between nDNA and cpDNA data for *Quercus fabri* is evident, facilitating effective nuclear gene flow through pollen [30]. The chloroplast-nuclear discordance exhibited in our study may be attributed to complex topography and limited distance of cytoplasmic gene flow via seed dispersal, and widespread recombination of the nuclear genome across the Southwest China indicating effective nuclear gene flow via pollen [31].

Possible reasons for the genealogical differentiation of the chloroplast data of populations near the west boundary of Yunnan possessing haplotypes of the east group of the *G. crenulata group* are firstly, that it corresponds with the “Tanaka line” identified by Tanaka in 1954 [32], indicating a split in the distribution of Citrus reticulata Blanco in Yunnan. This division likely influenced the early differentiation of the *G. crenulata* group. Secondly, changes in major water systems through the uplift of the Tibetan Plateau, such as diversions may also be relevant [33, 34]. For instance, the upper Jinsha, Yalong, and Dadu rivers, which once flowed into the ancient Red River, now exhibit a discontinuous structure. Geological events, roughly between the Miocene and Pleistocene, caused these alterations [35, 36], coinciding with the time of division of the *G. crenulata* group into two lineages (4.06 Ma, 95% HPD: 1.98–5.93 Ma, PP = 1.00). This suggests that the group was once continuously distributed in the valleys of ancient rivers, but present-day patterns hinder chloroplast gene flow between populations, which has led to significant genetic differentiation. Moreover, rivers like the Lancang, Nu, and Hong have maintained high levels of population dispersal and gene flow [36]. Similar patterns influenced by riverine changes are observed in other species such as *Terminalia franchetii* Gagnep., *Schizopygopsis malacanthus* Herzenstein, and *Incarvillea* arguta [37–39].

### The role of IBD in shaping the genetic structure of the *G. crenulata* group

We found that IBD rather than IBE influenced the genetic differentiation of the *G. crenulata* group, with a positive IBD correlation in the WC group as supported by both LCG and cpDNA data. Gene flow may follow patterns of IBE, whereby rates of gene flow rates higher among similar environments [40]. An IBD pattern is also often observed when gene flow is inversely proportional to geographic distance, limiting genetic variation in response to selection pressures primarily through natural dispersal and population size [41]. Genetic differentiation by IBD rather than IBE has been detected in many widespread species (e.g., *Echinacea angustifolia* DC [42], and *Mimulus guttatus* DC [43]), and this pattern has been suggested to facilitate colonization of new areas under conditions of constant disturbance [41, 44]. Because *G. crenulata* is a pioneer species, it can establish itself in newly created or recently disturbed environments after deforestation. These environments are more prone to continuous expansion due to human-induced forest degradation in subtropical China. This ongoing expansion process is likely to be driven more by geographic distance, resulting in genetic differentiation (IBD) rather than environmental adaptation (IBE).

We inferred a variant of the IBD pattern at a regional scale: the IBD pattern was only observed in the WC group and not in the EC group. IBD is often detected in populations of plants that tend to move shorter distances, resulting in a pattern of genetic divergence that increases significantly with geographic distance [45, 46]. In contrast to Eastern China, the topography of Western China is characterized by higher heterogeneity (e.g., a wider elevational range), facilitating genetic differentiation associated with phenological differences [47]. It is also characterized by more complex topographic barriers (e.g., high mountains and deep river valleys [36]), which, in the case of the *G. crenulata* group, have restricted dispersal and gene flow of the WC group. Accordingly, the absence of an IBD and IBE pattern in the EC group suggests higher gene flow and habitat homogeneity, possibly from recent colonization or extinction events and/or historical or biogeographic migration [41].

### Demographic history of the *G. crenulata* group

Our data revealed a population expansion in the WC group but not in the EC group. Ecological niche modeling indicated that the optimal potential range of the WC group (i.e., northwestern Yunnan and Southern Sichuan) had expanded from the MH period (ca. 6 kya) within the warm-period Holocene Thermal Maximum (5–11 kya). This period is characterized as a transition from arid to wet conditions [48, 49]. A similar expansion event occurring in Western China has been inferred in *Fokienia hodginsii* (Dunn) A.Henry & H.H.Thomas [50] and *Torreya fargesii* Franch. [51], the effects of glacial cycles could have been strongest in this area. The Hengduan Mountains serve as a migration corridor and refuge, facilitating rapid forest migration and providing safe havens during extreme climate events. As conditions improve, species can expand beyond these refuges [52]. Both climate-driven Holocene and geographical refugia facilitated the expansion of the WC group in this study. The ecological niche model also predicts contraction of the current optimal range of the *G. crenulata* group and a northward shift of its suboptimal range, strongly influenced by precipitation of the driest month, temperature annual range, and mean temperature of the wettest quarter. This potential range shift is consistent with the general trend of species shifting towards higher latitudes through global warming [53].

Based on the clustering results by STRUCTURE, the spatial genetic structure displayed a mosaic of clusters in the WC group. The mosaic population structure has been thought to likely result from stochastic short-range dispersal of individuals [54], also indicated by IBD detected in this group. Our study revealed historical gene flow of the *G. crenulata* group from east to west based on the LCG data (Nm (EC to WC) = 88.87 >  > Nm (WC to EC) = 2.94). Asymmetrical gene flow from east to west was also found as occurring in widespread Chinese species such as *Juglans cathayensis* Dode [55], *Arnebia szechenyi* Kanitz [56], and *Q. acutissima * [57]. This asymmetry was postulated as facilitated by the East Asian monsoon, hybrid incompatibility, and wind-mediated pollen dispersal. Species of *Gaultheria* can be pollinated by insects and wind [58, 59]. In our study, east-to-west gene flow of the *G. crenulata* group may be enhanced by pollen-mediated movement influenced by the East Asian monsoon system, the significant role of which in shaping population genetic structure has also been demonstrated in plants such as *Primulina* Hance [60] and *Quercus chenii* Nakai [61].

According to the DIYABC-RF analysis, the narrowly endemic species *G. pingbienensis* (DWS), *G. luchunensis* (YLC), *G. wuliangshanensis* (JD), and *G. mangshanensis* (NMS) were inferred to have diverged from their ancestral population ca. 0.1 (0.103–0.140) million years ago during the Pleistocene refugium period (Fig. 3-C). Previous studies have reported the existence of multiple refugia during the Quaternary ice age in this region [62]. The presence of these refugia facilitated the divergence of surviving species through long-distance isolation [12, 63]. Additionally, we found that distinct haplotypes of these four species successively emerged during the Pleistocene (Fig. 4), indicating significant divergence events during Pleistocene glaciation. These four species thrive in undisturbed high mountain ridges and moist slope forest environments with high endemism. *Gaultheria pingbienensis*, *G. luchunensis*, *G. wuliangshanensis* are distributed in the Wuliang Mountains of the Central Yunnan Plateau, and *G. mangshanensis* is distributed in the Nanling Mountains of the Chinese southern hills. Conversely, *G. crenulata* prefers disturbed secondary forests with full or partial sun exposure [22]. The isolation of populations on separate mountains, which occurred during warm periods when the species followed its optimal climate towards higher elevations, was probably caused by ancient glacial cycles, which occurred from over 1 Ma to about 0.1 Ma BP [64]. Related studies found that during the last interglacial period (0.1 Ma), summer monsoon rains and temperatures increased in vast areas across Asia [65], which may have also facilitated the divergence of these four species. Furthermore, the isolation of *G. crenulata* of the WC group and EC group at 0.617 Ma BP, although more recent compared to the uplift of the Himalayan–Tibetan Plateau and the formation of the staircase topography since the Late Miocene times [66], has been detected in many other species as well. For example, the divergence time between the eastern and western lineages of *Liriodendron chinense* (Hemsl.) Sarg is estimated to be ca. 0.433 Ma [67] and Asteropyrum J. R. Drumm. & Hutch. is dated to 1.2 Ma [68]. Our data indicate that the east–west lineage split is a gradual process of geographic isolation and allopatric speciation.

### Population genetic differentiation and conservation in the secondary forests of the subtropical China

The significant genetic differentiation observed in the *Gaultheria crenulata* group along an east–west axis in subtropical China is consistent with patterns seen in other tree species in the same region, such as *Quercus acutissima * [4]., *Q. fabri* [15], and *L. chinense * [69]. This differentiation is primarily attributed to historical geographic and climatic shifts during the Pliocene. Conversely, tree species in the secondary forests, such as *Camellia oleifera* Abel and *Liquidambar formosana* Hance, did not display genetic differentiation along an east–west axis [70]. Instead, they showed low genetic diversity within populations and high genetic differentiation among populations, largely due to anthropogenic disturbance and habitat fragmentation [16, 70]. Anthropogenic activities, such as deforestation for agriculture, urbanization, and logging, have led to habitat loss and fragmentation [71], gexacerbating genetic isolation among populations [72]. The conversion of natural forests into plantations and secondary forests has created barriers to gene flow, resulting in genetic differentiation among isolated populations of the pioneer tree *Cecropia hololeuca* Miq. [73]. Human activities, such as changes in population density, cropland use, and irrigated rice area, are significantly linked to the demographic history of *Litsea elongata* (Wall. ex Ness) Benth. & Hook. f [74].These disturbances, combined with climate change, pose significant threats, including reduced habitat range, increased risk of extinction, and loss of genetic diversity [75].

In our study, the shrubs of the *G. crenulata* group exhibited low genetic variation within populations (*H*_S_ = 0.091) and moderate levels of genetic variation among populations (*H*_T_ = 0.34). This indicates that although there is some genetic diversity within populations, there is also significant genetic differentiation among populations. Similarly, *Pinus armandii* Franch, a companion tree species to the *G. crenulata* group, showed lower levels of genetic diversity in planted populations, highlighting the broader challenge of maintaining genetic diversity in the plant species of the region [76].

Our field observations indicate declines in populations, particularly in regions with frequent specimen records from the past, such as Mount Emei in Sichuan Province. This decline may be attributed to fluctuations in temperature and precipitation over the past decades [77]. Additionally, populations of the *G. crenulata* group are usually clonal, reproducing vegetatively through rhizomes [78]. The populations in montane central and northeastern Yunnan have been removed by local minorities or pharmacy companies which has also substantially reduced the biomass of this medicinal plant.

The significant genetic differentiation observed in the *G. crenulata* group, influenced by both historical factors and contemporary anthropogenic disturbance, underscores the importance of conservation efforts to preserve this species. Anthropogenic activities, including deforestation, urbanization, and habitat fragmentation, have contributed to the genetic differentiation and decline of these species. Our study predicts that as climate warms, the potential distribution of the *G. crenulata* group will continuously shrink, especially in optimal areas like Chongqing, Hubei, and Northern Guizhou (Fig. 5). Thus, conservation strategies should address the complex interplay of historical and environmental factors, as well as anthropogenic activities, to ensure the genetic diversity and future survival of the *G. crenulata* group.

## Conclusions

The low genetic diversity and IBD pattern detected in the WC group of *G. crenulata* for gene differentiation can be inferred to result from the disturbance of human activities. The region covering central and northern Yunnan and southern Sichuan is characterized by the potentially optimal range with highest genetic diversity of the *G. crenulata* group. Such a region possesses high ability to withstand adverse environmental conditions and plays a vital role in promoting ecosystem restoration [79]. From a protection perspective, it is crucial to enhance conservative efforts in this region and promote habitat restoration, to ensure sustainable utilization of the *G. crenulata* group.

## Materials and methods

### Field sampling

The sampling design encompassed the entire geographic range of the *Gaultheria crenulata* group in subtropical China based on a recent taxonomic revision for the group [22]. To fully cover the geographic extent of our focal group, we consulted nearly all digitized specimens of the group in the Chinese Virtual Herbarium Database (https://www.cvh.ac.cn/), with a total of 1,434 specimens (1924–2016). Based on the locality information from these specimens, we collected 1,435 individuals from 89 natural populations (Fig. 1), including 85 from *G. crenulata* (widely distributed across the entirety of the subtropical China), one (YLC) from *G. luchunensis* (Luchun, Yunnan), one (DWS) from *G. pingbienensis* (Pingbian, Yunnan), one (JD) from *G. wuliangshanensis* (Jingdong, Yunnan), and one (NMS) from *G. mangshanensis* (Mangshan, Hunan). The distribution of the latter four species overlapped that of *G. crenulata* but are narrowly endemic, with only one locality known each. Our sampling covered 70% of the counties with the majority distributed across ten provinces/municipalities south of the Yangtze River in China. The number of individuals sampled from each population varied because of differences in population size and local occurrence frequency. Sample sizes ranged from six to 29 per site, with the exception of the EMS population (where only one individual was found) and the DWY population (where only two individuals were found). Despite their limited samples sizes, we deemed it important to include these populations in our analysis because they cover geographical areas not represented by other populations. The average sample size across all populations was 16 (Table 1). Within-population individuals were collected at least 30 m from one another to reduce spatial autocorrelation. Lu Lu undertook the formal identification of the plant material used in our study. Samples consisted of leaves dried with silica-gel desiccant. Specimen vouchers for each population were deposited in the Herbarium of the Kunming Institute of Botany, Chinese Academy of Sciences (KUN).

### DNA amplification and sequencing

DNA was extracted from ca. 200 mg of each leaf sample with the cetyltrimethyl ammonium bromide protocol [80]. Two chloroplast DNA fragments (*rpl*33-*psa*J and *trn*L-*rpl*32) and the two low-copy nuclear genes *AAT* and *LOC* (LCG) were employed (Table S8). PCR conditions were generally conducted as follows: 94 °C for four min, followed by 35 amplification cycles each at 94 °C for one min, annealing at 54–60 °C for 45 s, 72 °C for one min, and a final elongation at 72 °C for 10 min. The purified gene products were direct-sequenced with the Sanger method by using amplification primers and BigDye on an ABI 3730 capillary sequencer (Applied Biosystems, foster City, California, USA). Nuclear-gene PCR products that failed with direct sequencing were cloned into the pUC57-Kan vector (GenScript) for isolation of sequences. Eight single colonies per individual were selected randomly for colony PCR, and colonies containing target-size amplicons were sequenced with universal primers M13F and M13R (Sangon Biotech Co., Ltd., Shanghai, China). Sequences were edited and assembled with Sequencher [81] and Se-Al (University of Oxford, http://evolve.zoo.ox.ac.uk/software/seal/). All sequence data were deposited in GenBank (under accession numbers OR327752-OR333491).

### Genetic diversity and differentiation statistics

Heterozygous sequences of the LCG were phased by using DnaSP 6.12 [82] with 1,000 runs, and a burn-in of 100, then analyzed with SPADS 1.0 [83] for calculating allelic richness (*A*_R_), nucleotide diversity (*π*), and interpopulation genetic differences (*N*_ST_ and *G*_ST_). cpDNA sequences were analyzed with DnaSP 6.12 for haplotype diversity (*H*_d_) and π, and with PERMUT 1.0 [84] for calculating genetic diversity (*H*_T_), intrapopulation genetic diversity (*H*s), *N*_ST_, and *G*_ST_. Arlequin 3.11 [85] was used to calculate the coefficient of genetic differentiation (*F*_ST_) among populations and estimate variation within populations.

### Population genetic structure and migration

Based on our preliminary genetic analysis with STRUCTURE 2.3.4 [86], we divided the 89 population samples into the Western China (WC) group and the Eastern China (EC) group, separating the second-step and third-step regions. STRUCTURE was used to investigate the population structure of the LCG data based on an admixture model with correlated allele frequencies. The analysis involved 100,000 MCMC steps, with a burn-in of 50,000 and K values ranging from 1 to 10, repeated 20 times. We employed HARVST (http://taylor0.biology.ucla.edu/structureHarvester/) to calculate the posterior probability lnP (D) and its change rate ΔK to determine the best grouping [87]. To visualize the genetic similarity among populations, Principal coordinates analysis (PCoA) was performed with GenAlEx 6.54 [88].

For the network evolutionary relationships based on cpDNA data, we constructed haplotypes using the median linkage method in NETWORK 10.2 [89]. We also mapped the geographic distribution of the STRUCTURE grouping results and cpDNA haplotypes for each population using the ArcMap package in ArcGIS 10.2 (ESRI Inc, Redlands, California, USA). The Bayesian search strategy in MIGRATE 3.6.8 [90] was used with the LCG data to estimate potential historical gene flow (Nm) between the two groups. We calculated Θ = 4Nμ (N, effective population size; μ, mutation rate per generation) and *M* = m/μ (m, migration rate per generation) using the effective number of migrations. The parameters used were long-chains = 1, long-inc = 100, long-sample = 500,000, and burnin = 10,000. The initial values of Θ and *M* were derived from the *F*_ST_ values. We used SplitsTree 4.14.8 [91] to infer a neighbor net plot, visualizing the relationships among species from *F*_ST_ values of the nuclear and chloroplast data.

### Population historical dynamics and divergence time estimates

We calculated Tajima’s *D* test [92] and Fu & Li’s D* test [93] to assess neutral evolution for both the LCG and cpDNA data. Mismatch distribution analysis was performed with Arlequin 3.11 by calculating Harpending’s raggedness index (HRag) and the sum of deviation squares (SSD) with 500 repetitions [94, 95] for either the overall data (populations) or for the two groups.

Colonization pathways based on the LCG data were analyzed by DIYABC Random Forest 1.0 (the DIYABC-RF analysis) [96]. Model construction was based on both taxonomic delimitation [22] and the geographical ranges of the populations (Fig. 1, (Fig. S1). The populations of the *Gaultheria crenulata* group were therefore classified into six groups: *G. crenulata* from the WC group (Pop1; denoted WC), *G. crenulata* from the EC group (Pop2; EC), *G. wuliangshanensis* (Pop3; JD), *G. pingbienensis* (Pop4; DWS), *G. luchunensis* (Pop5; YLC), and *G. mangshanensis* (Pop6; NMS). In addition, the results of population differentiation were used to pool genomically similar geographical sites and help guide the building of the models (Fig S3). We used the “random forest” method to classify the most likely scenario and estimate parameters, based on 20,000 and 50,000 simulations, respectively. We conducted 1,000 random forest trees and set uniform distributions as priors for all parameters. Generations time was based on the average five-year reproductive maturity period for *Gaultheria* [26] (Tables S4–S6).

Because of the low mutation rate of cpDNA data, the DIYABC-RF analysis could not be applied. We therefore used BEAST 2.4.2 [97] to estimate the haplotype divergence time for cpDNA differentiation. *Gaultheria fragrantissima* Wall. was selected as outgroup, and the calibrated Yule prior, strict clock model, and normal-distributed rate set were used. The program jModelTest 2.1.1 [98] yielded HKY + I + G model as the best model. Tracer 1.6 [99] was employed to validate the effective sample sizes (ESS > 200) for all prior and posterior distributions. As a secondary calibration point, we used the stem node age of the *G. crenulata* group [100] with a priori mean = 4.06 Ma, SD = 1 Ma, and 95% CI = 1.7–7.59 Ma on the fossil-calibrated phylogeny of the tribe Gaultherieae. The MCMC run consisted of 1 × 10 [7] generations, with sampling occurring every 1000 generations. The first 25% of samples were discarded as burn-in. Tree files were displayed with Figtree 1.4.3 (http://tree.bio.ed.ac.uk/software/figtree/).

### IBD and IBE analyses

To investigate the influence of geography and/or environment on genetic differentiation, we tested isolation-by-distance (IBD) and isolation-by-environment (IBE) for the overall (populations) and for the WC/EC groups. For the LCG data, we assessed genetic distance using the values of pairwise differentiation (*F*_ST_) and *F*_ST_/ (1 − *F*_ST_) calculated with Arlequin. For cpDNA data, we assessed genetic distance through F_ST_ and nucleotide divergence (*D*_XY_) using DnaSP. We characterized major environmental variations using bioclimatic variables obtained from WorldClim (http://worldclim.org) via Principal Component Analysis (PCA) with PC1 and PC2. We computed environmental and geographical distances using “vegdist” in the “vegan” package of R 4.05 (https://www.r-project.org/) and GenAlEx 6.54, respectively. To assess contributions, we used multiple matrix regression with randomization (MMRR) analysis to evaluate geographic distance and environmental variables [101]. We estimated correlations between genetic distance and effects from either geographic distance (with environmental variables removed) or environmental variables (with geographical distances removed) using partial Mantel tests. All analyses were conducted in R with 10,000 iterations.

### Ecological niche modeling

Climate data of the Last Glacial Maximum (LGM; ca. 21 kya), mid-Holocene (MH; ca. 6 kya), current (1950–2000), and future (2070) were obtained from WorldClim, each period containing 19 bioclimatic variables with a 2.5 arc-minute spatial resolution. We simulated the LGM, MH, and current data using the CCSM4 model, whereas the future data was based on the RCP 8.5 scenario [102]. Considering the genetic structure may affect the results, we used GPS data of the *G. crenulata* group to generate species distribution models for the WC and EC groups, respectively. We identified correlated environmental variables using Pearson’s correlation coefficient algorithm, with a cutoff of |0.8| to prevent model overfitting [103]. Selected bioclimatic variables (bio1, bio2, bio5, bio6, bio7, bio8, bio12, bio14, and bio18) were imported into MaxEnt 3.3.3, with 75% of the data used for training and 25% for calibration; defaults were used for the other parameters. Model validation was replicate for 10 times independently, maximum iterations = 5000 with a convergence threshold 10^−5^. We evaluated each model using the area under the curve (AUC) of the receiver operating characteristic [104]. Maxent files were exported as a logistic output layer consisting of a grid map with ArcGIS.

### Supplementary Information

Supplementary Material 1.

Supplementary Material 2.

Supplementary Material 3.

Supplementary Material 4.

## Data Availability

Data is provided within the manuscript or supplementary information files.

## Abbreviations

*A*_R_: Allelic richness
AUC: The area under the curve
cpDNA: Chloroplast DNA
EC: Eastern China
ESS: Effective sample sizes
*F*_ST_: Genetic differentiation
*H*d: Haplotype diversity
*H*_S_: Intrapopulation genetic diversity
*H*_T_: Genetic diversity
IBD: Isolation-by-distance
IBE: Isolation-by-environment
LCG: Low-copy nuclear genes
LGM: Last Glacial Maximum
MH: Mid-Holocene
Nm: Gene flow
PCoA: Principal coordinates analysis
SSD: The sum of deviation squares
WC: Western China
*π*: Nucleotide diversity
