# Supplementary figures and images for: Population differentiation and dynamics of five pioneer species of Gaultheria from the secondary forests in subtropical China
Source: BMC Plant Biol. 2024 Jun 8;24:516. doi: 10.1186/s12870-024-05189-z (PMC11161945; doi:10.1186/s12870-024-05189-z)

(b)

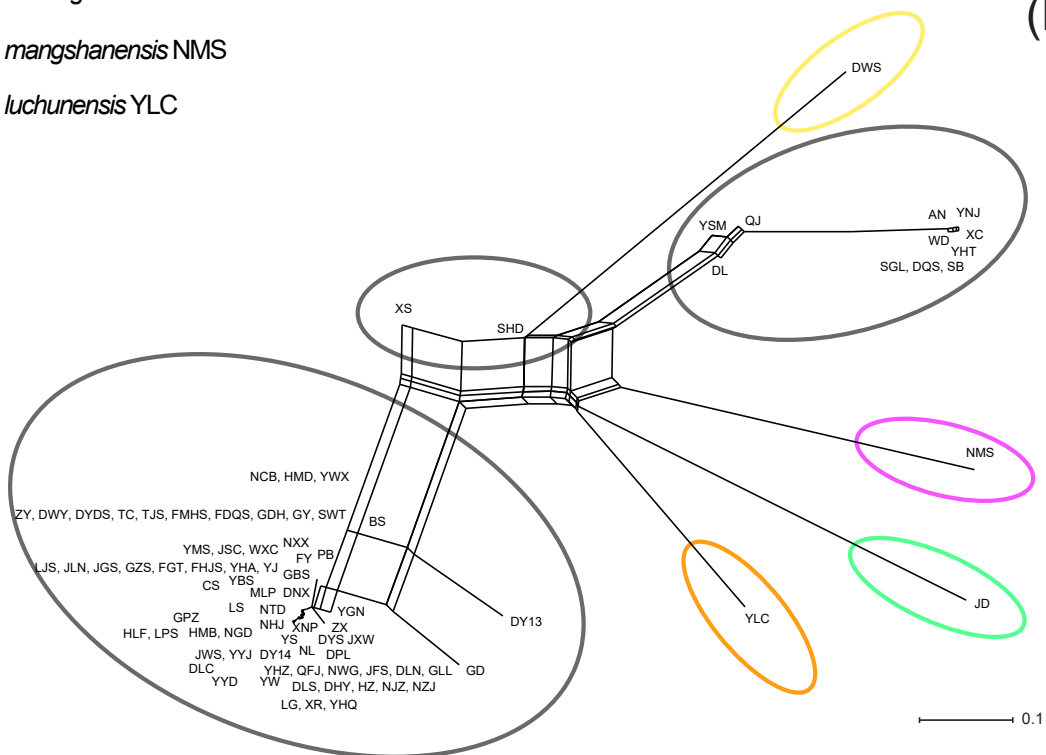

Fig.S2

Supplement: Supplementary file 2 — Supplementary Material 2. [file 12870_2024_5189_MOESM2_ESM.pdf]

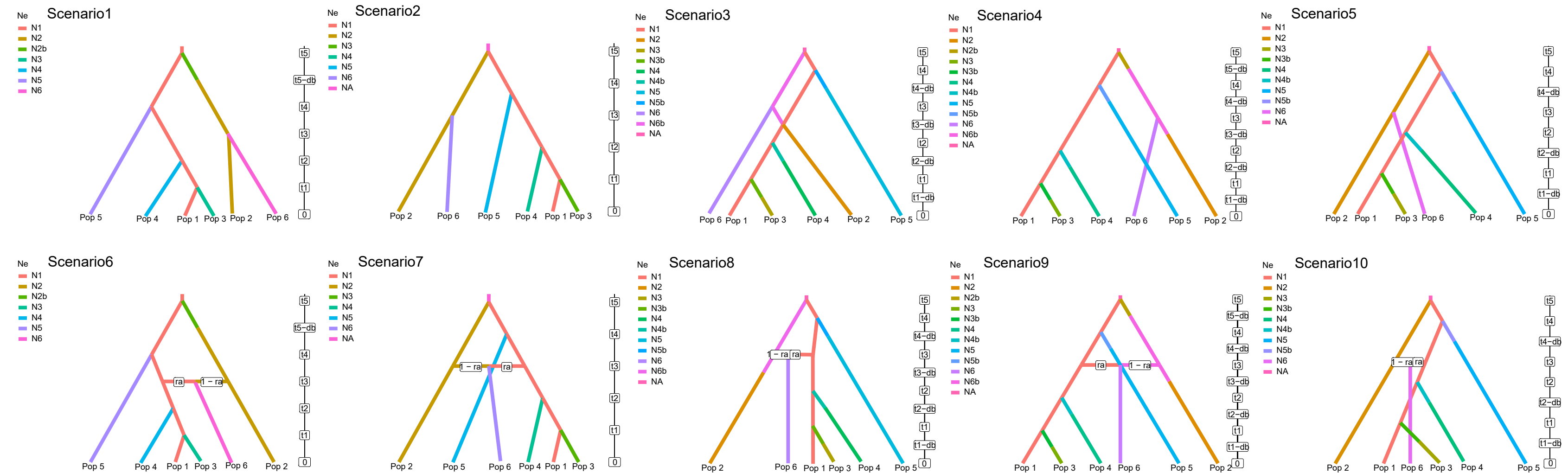

Fig. S3

Supplement: Supplementary file 3 — Supplementary Material 3. [file 12870_2024_5189_MOESM3_ESM.pdf]
