# Supplementary material for: Population differentiation and dynamics of five pioneer species of Gaultheria from the secondary forests in subtropical China
Source: BMC Plant Biol. 2024 Jun 8;24:516. doi: 10.1186/s12870-024-05189-z (PMC11161945; doi:10.1186/s12870-024-05189-z)
Supplement: Supplementary file 4 — Supplementary Material 4. [file 12870_2024_5189_MOESM4_ESM.docx]

**Supplementary legends**

**Fig. S1** Bayesian cluster analysis with STRUCTUR for 1435 individuals across 89 populations of the *G. crenulata* group. **(a)** Delta K statistics; **(b)** Mean estimated Log probability of data for different cluster numbers (K); **(c)** STRUCTURE bar plot (K values 1–10) based on probabilities, with black lines separating populations.

**Fig. S2** Neighbor-net based on a genetic distance (F_ST_) matrix among 89 populations from the *G. crenulata* group. **(a)** The network tree based on LCG data; **(b)** The network tree based on cpDNA data.

**Fig. S3** Population divergence of the *G. crenulata* group based on the DIYABC-RF analysis (prior on admixture proportion: admixture rate of 0.05–0.95 with a uniform distribution, default setting). There are two evolutionary hypotheses demonstrated by ten scenarios in total. **Hypothesis 1** (indicated by scenarios 1 through 5) yielded no admixture events, and Pop2 and Pop6 split from a common ancestor. **Hypothesis 2** (indicated by scenarios 6 through10) yielded a model divergence between Pop1 and Pop2 with a single admixture event to form Pop6. Demographic and historical parameters include six effective population sizes N1, N2, N3, N4, N5, N6 (for populations 1, 2, 3, 4, 5, and 6 respectively) and five divergence time events (t1, t2, t3, t4, t5) and a given number of generation (here db for each population). For the scenarios with admixture, the parameter “ra” corresponds to the proportion of genes of a given source population entering the admixed population.

**Table S1** Estimates of gene flow (Nm) between two groups (WC and EC) in the *G. crenulata* group based on LCG data.

**Table S2** Results of neutrality test (Tajima's D, Fu and Li’s D* tests) and mismatch distributions analysis for the two lineages identified in the STRUCTURE analysis. Sum of Squared deviation (SSD), Harpending’s Raggedness index (HRag), Tajima’s D, and Fu and Li’s D*.

**Table S3** The accuracy of model predictions and the classification error rate as determined by the random forest (RF) algorithm. RF generates predictions using 1,000 trees and a trained set of 20,000 simulated predictor variables (summary statistics). The demographic model serves as the response variable for RF. The proportion of accurately predicted demographic models is highlighted in bold.

**Table S4** The proportion of accurately predicted models and the classification error rate, as estimated by the random forest (RF) algorithm. RF predictions are generated from 1,000 trees and a trained set of 50,000 simulated predictor variables (summary statistics). The demographic model serves as the response variable for RF. The proportion of accurately predicted demographic models is highlighted in bold.

**Table S5** For model selections and predictions, the random forest (RF) algorithm produces predictions by aggregating votes from 1,000 trees. These predictions encompass the outcomes of the selected model and the posterior probability associated with the chosen model (i.e., scenario).

**Table S6** Prior distributions parameters for the *G. crenulata* group in Approximate Bayesian Computation and the corresponding posterior distributions for each parameter under the priority scenario.

**Table S7** The contributions of the nine environment variables in species distribution modeling at the present for the *G. crenulata* group in subtropical China.

**Table S8** Basic information for two cpDNA primers and two self-developed nuclear low copy gene primers in the *G. crenulata* group.

**Supplementary tables**

**Table S1** Estimates of gene flow (Nm) among two groups within the *G. crenulata* group based on LCG data.

| Group | Θ*i* | Nm (WC)  →*i* | Nm (EC)  →*i* |
| --- | --- | --- | --- |
| WC | 0.09664 | NA | 88.87 |
| EC | 0.00637 | 2.94 | NA |

Abbreviations: WC, western China group; EC, eastern China group.

**Table S2** Results of neutrality test (Tajima's *D*, Fu and Li’s *D** tests) and mismatch distributions analysis for the two lineages identified in the analysis of STRUCTURE analysis, Sum of Squared deviation (SSD), Harpending’s Raggedness index (HRag), Tajima’s *D*, and Fu and Li’s *D**.

| Region | Neutrality test | | Mismatch distribution | |
| --- | --- | --- | --- | --- |
|  | Tajima’s *D* | Fu and Li’s *D** | SSD | HRAG |
| Overall LCGs | -1.8803** | 3.7249** | 0.0064 | 0.0077 |
| Overall cpDNA | -1.3975* | -3.8003** | 0.0565 | 0.4529 |
| WC LCGs | -1.7758** | 3.4592** | 0.0036 | 0.0028 |
| WC cpDNA | -0.9904 | -4.1559** | 0.1550 | 0.3857 |
| EC LCGs | -0.9113 | 1.3631 | 0.0528 | 0.0685 |
| EC cpDNA | -1.2438 | 1.2038 | 0.0054 | 0.7976 |

Abbreviations: WC, western China group; EC, eastern China group; *, *P* < 0.05; **, *P* < 0.02.

**Table S3** The accuracy of model predictions and the classification error rate as determined by the random forest (RF) algorithm. RF generates predictions using 1,000 trees, utilizing a trained set of 20,000 simulated predictor variables (summary statistics). The demographic model serves as the response variable for RF. The proportion of accurately predicted demographic models is highlighted in bold.

| Simulated | Predicted model | | Classification |
| --- | --- | --- | --- |
| model | without admixture | with admixture | error rate |
| NA | **0.837** | 0.163 | 0.163 |
| WA | 0.063 | **0.937** | 0.063 |

Abbreviations: NA, nonadmixed; WA, with admixture.

**Table S4** The proportion of accurately predicted models and the classification error rate, as estimated by the random forest (RF) algorithm. RF predictions are generated from 1,000 trees using a trained set of 50,000 simulated predictor variables (summary statistics). The demographic model serves as the response variable for RF. The proportion of accurately predicted demographic models is highlighted in bold.

| Simulated  model | Predicted model | | | | | | | | | | Classification error rate |
| --- | --- | --- | --- | --- | --- | --- | --- | --- | --- | --- | --- |
|  | NA1 | NA2 | NA3 | NA4 | NA5 | WA1 | WA2 | WA3 | WA4 | WA5 |  |
| NA1 | 0.309 | 0.222 | 0.034 | 0.041 | 0.222 | 0.060 | 0.045 | 0.012 | 0.007 | 0.048 | 0.691323 |
| NA2 | 0.220 | 0.278 | 0.030 | 0.037 | 0.253 | 0.050 | 0.065 | 0.009 | 0.005 | 0.053 | 0.721956 |
| NA3 | 0.054 | 0.055 | 0.358 | 0.320 | 0.056 | 0.014 | 0.009 | 0.064 | 0.060 | 0.010 | 0.641524 |
| NA4 | 0.065 | 0.057 | 0.323 | 0.360 | 0.058 | 0.013 | 0.013 | 0.055 | 0.046 | 0.010 | 0.640129 |
| NA5 | 0.213 | 0.263 | 0.037 | 0.035 | 0.276 | 0.034 | 0.057 | 0.011 | 0.010 | 0.064 | 0.723691 |
| WA1 | 0.029 | 0.025 | 0.007 | 0.005 | 0.014 | 0.331 | 0.260 | 0.042 | 0.052 | 0.235 | 0.668605 |
| WA2 | 0.024 | 0.035 | 0.008 | 0.010 | 0.030 | 0.241 | 0.298 | 0.028 | 0.042 | 0.284 | 0.702203 |
| WA3 | 0.005 | 0.003 | 0.014 | 0.019 | 0.002 | 0.062 | 0.051 | 0.402 | 0.381 | 0.061 | 0.598352 |
| WA4 | 0.009 | 0.004 | 0.008 | 0.008 | 0.007 | 0.075 | 0.068 | 0.362 | **0.395** | 0.064 | **0.605114** |
| WA5 | 0.023 | 0.026 | 0.012 | 0.007 | 0.034 | 0.237 | 0.269 | 0.040 | 0.039 | 0.313 | 0.687209 |

Abbreviations: NA, nonadmixed; WA, with admixture.

**Table S5** For model selections and predictions, the random forest (RF) algorithm produces predictions by aggregating votes from 1,000 trees. These predictions encompass the outcomes of the selected model and the posterior probability associated with the chosen model (i.e., scenario).

| Votes  NA1 | Votes  NA2 | Votes  NA3 | Votes  NA4 | Votes  NA5 | Votes  WA6 | Votes  WA7 | Votes  WA8 | Votes  WA9 | Votes  WA10 | selected model | posterior probability |
| --- | --- | --- | --- | --- | --- | --- | --- | --- | --- | --- | --- |
| 61 | 70 | 78 | 87 | 78 | 68 | 93 | 147 | **192** | 126 | 9 | **0.444** |

**Table S6** Prior distributions parameters for the *G. crenulata* group in Approximate Bayesian Computation and the corresponding posterior distributions for each parameter under the priority scenario.

| Parameter | With admixture Scenario 9 | | | | |
| --- | --- | --- | --- | --- | --- |
| Effective population size | Priori | Expectation | Median | Quantile_0.05 | Quantile_0.95 |
| N1 | 1-500000 | 408000.0 | 419700.0 | 276285.0 | 492609.0 |
| N2 | 1-500000 | 221378.0 | 184932.0 | 42070.7 | 465698.0 |
| N3 | 1-500000 | 10265.6 | 5605.7 | 662.3 | 38964.7 |
| N4 | 1-50000 | 22765.9 | 20901.0 | 6984.5 | 45806.2 |
| N5 | 1-50000 | 26515.1 | 25808.2 | 7530.1 | 47380.0 |
| N6 | 1-50000 | 25282.8 | 24383.0 | 7635.9 | 46264.4 |
| NA | 1-500000 | 130570.0 | 99527.8 | 6662.8 | 406419.0 |
| N2b | 1-50000 | 24778.7 | 25311.0 | 1708.0 | 47662.8 |
| N3b | 1-50000 | 23502.5 | 23218.0 | 1084.1 | 47128.6 |
| N4b | 1-50000 | 23755.2 | 22946.0 | 1918.7 | 47994.5 |
| N5b | 1-50000 | 23755.8 | 23156.1 | 2720.8 | 47192.0 |
| N6b | 1-50000 | 27064.5 | 26361.6 | 7003.7 | 47550.4 |
| Time of events |  |  |  |  |  |
| T1 | 10-50000 | 31966.5 | 33312.8 | 9317.0 | 48412.2 |
| T2 | 10-50000 | 31393.5 | 32031.9 | 11702.0 | 48113.0 |
| T3 | 10-50000 | 26435.0 | 26352.2 | 5229.0 | 47764.0 |
| T4 | 10-50000 | 26695.4 | 25978.8 | 5362.5 | 47754.7 |
| T5 | 10-1000000 | 185787.0 | 128866.0 | 32653.3 | 510468.0 |
| db | 1-10000 | 5205.0 | 5340.0 | 450.0 | 9585.8 |
| ra | 0.0001-0.9999 | 0.4283 | 0.3976 | 0.1062 | 0.8332 |

**Table S7** The contributions of the nine environment variables when conducting species distribution modeling at the present for the *G. crenulata* group in subtropical China.

| Variable | Coded means | WC group percent contribution (%) | WC group permutation importance (%) | EC group percent contribution (%) | EC group permutation importance (%) |
| --- | --- | --- | --- | --- | --- |
| Bio1 | Annual Mean Temperature | 20 | 7.4 | 2.8 | 0.6 |
| Bio2 | Mean Diurnal Range (Mean of monthly (max temp - min temp)) | 0.7 | 4.4 | 0.4 | 2.8 |
| Bio5 | Max Temperature of Warmest Month | 7.6 | 2.9 | 8.9 | 3.4 |
| Bio6 | Min Temperature of Coldest Month | 8.1 | 48.2 | 5.9 | 65.6 |
| Bio7 | Temperature Annual Range (BIO5 - BIO6) | 16.7 | 22 | 0.9 | 11.3 |
| Bio8 | Mean Temperature of Wettest Quarter | 2.9 | 0.8 | 8.3 | 0.2 |
| Bio12 | Annual Precipitation | 3.4 | 9.8 | 3.4 | 5.5 |
| Bio14 | Precipitation of Driest Month | 33.9 | 3.4 | 68.3 | 7.8 |
| Bio18 | Precipitation of Warmest Quarter | 6.7 | 1.2 | 1 | 2.8 |

**Table S8** Basic information for two cpDNA primers and two self-developed nuclear low copy gene primers in the *G. crenulata* group.

| Region | Primer sequence (5'to 3') | Aligned length (bp) | Annealing temp(°C) | References |
| --- | --- | --- | --- | --- |
| *rpl*33*-psa*J | F: TCCGGATGCGTTAACATTCCCCTT | 669 | 60 | Li et al., 2020 |
|  | R: CCTTGGAAGGGTAACACACAGGTGC |  |  |  |
| *trn*L*-rpl*32 | trnL: CTGCTTCCTAAGAGCAGCGT | 568 | 56 | Li et al., 2020 |
|  | rpL32: CAGTTCCAAAAAAACGTACTTC |  |  |  |
| *AAT* | F253: CACAGCTGGAAGCACATCAC | 580 | 60 | Gong and Gong, 2016 |
|  | R581: GCACTTTTAGCATGGAAGGC |  |  |  |
| *LOC* | F：AGTAATTGTGGGCTTGGATT | 759 | 54 | Li et al.,2023 |
|  | R：TAAGGGTAACACCTTGCATCT |  |  |  |
