# Supplementary material for: Population differentiation and dynamics of five pioneer species of Gaultheria from the secondary forests in subtropical China
Source: BMC Plant Biol. 2024 Jun 8;24:516. doi: 10.1186/s12870-024-05189-z (PMC11161945; doi:10.1186/s12870-024-05189-z)
Supplement: Supplementary file 1 — Supplementary Material 1. [file 12870_2024_5189_MOESM1_ESM.pdf]

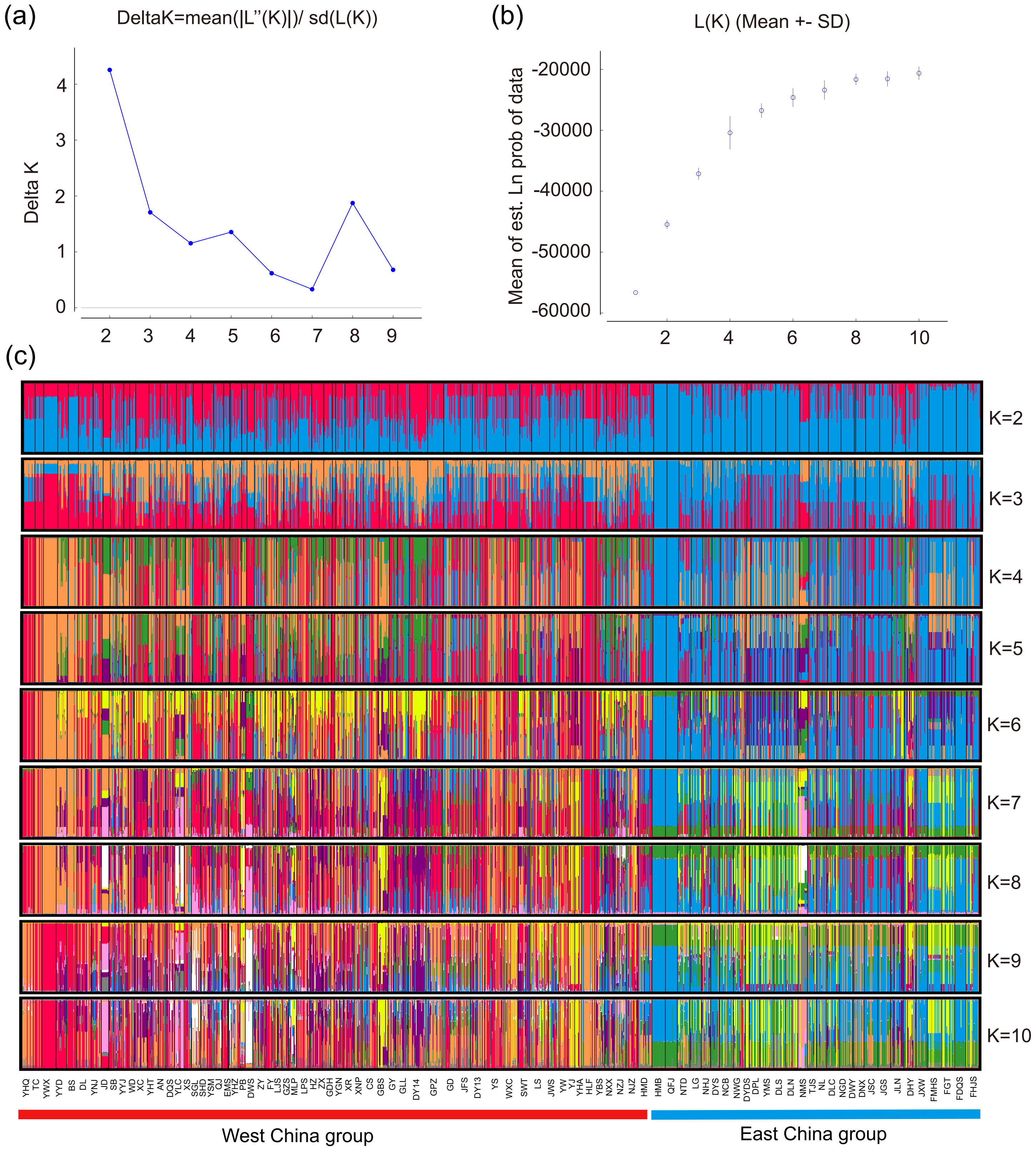
(a)
DeltaK=mean(|L''(K)|)/ sd(L(K))
Delta K
0
1
2
3
4
2
3
4
5
6
7
8
9
(b)
L(K) (Mean +- SD)
Mean of est. Ln prob of data
-20000
-30000
-40000
-50000
-60000
2
4
6
8
10
(c)
K=2
K=3
K=4
K=5
K=6
K=7
K=8
K=9
K=10
YHQ TC YWX YYD BS DL YNJ JD SB YYJ WD XC YHT AN DQS YLC XS SGL SHD YSM QJ EMS YHZ PB DWS ZY FY LJS GZS MLP LPS HZ ZX GDH YGN XR XNP CS GBS GY GLL DY14 GPZ GD JFS DY13 YS WXC SWT LS JWS YW YJ YHA HLF YBS NXX NZJ NJZ HMD HMB QFJ NTD LG NHJ DYS NCB NWG DYDS DPL YMS DLS DLN NMS TJS NL DLC NGD DWY DNX JSC JGS JLN DHY JXW FMHS FGT FDQS FHJS
West China group
East China group

Fig. S1
